# A mining code for regulating lunar water ice mining activities

**DOI:** 10.1073/pnas.2321079121

**Published:** 2024-12-16

**Authors:** Kevin M. Hubbard, Linda T. Elkins-Tanton, Tanja Masson-Zwaan

**Affiliations:** ^a^School of Earth and Space Exploration, College of Liberal Arts and Sciences, Arizona State University, Tempe, AZ 85287; ^b^International Institute of Air and Space Law, Institute of Public Law, Leiden University, Leiden 2311 ES, The Netherlands

**Keywords:** Moon, International Seabed Authority, water, space resources, space policy

## Abstract

Despite the increasing number of space launches, growth of the commercial space sector, signing of the Artemis Accords, maturation of space mining technologies, the emergence of a regulatory environment through domestic legislation, and a comprehensive body of international law, an intergovernmental governing authority has yet to be established to manage mining activities on the Moon. We developed a Lunar Mining Code and mapping tool to regulate and manage prospecting and exploration activities for water ice at the Moon’s poles. The Lunar Mining Code is composed of a notification system to manage prospecting, a contract system for issuing exploration licenses to allotted areas on the Moon, and best mining practices and principles to promote equal access and safeguard the lunar environment.

Even before the Apollo era, scientists hypothesized that water ice, useful for rocket propellant, oxygen, and drinking water might be present in mineable quantities on the Moon (1). Since then, multiple lines of evidence demonstrate that the Moon’s poles do indeed contain water ice in cold traps (2–8). In fact, the plume produced during NASA’s Lunar Crater Observation Satellite Mission contained 5.6 ± 2.9 wt. % water ice (9, 10), suggesting water resources that would dramatically reduce the cost of space exploration. The rising number of space launches (11), development of space resources technologies (12), growth of the commercial space sector, signing of the Artemis Accords (13), the emergence of a regulatory environment through domestic legislation (14), and calls for a space resources governance framework (15) demonstrates that space mining is on the horizon. Moreover, the United Nations Committee on the Peaceful Uses of Outer Space set up a Space Resources Working Group (16) and proposed an action team to assist in future technical and operational issues faced by lunar operators (17). The Treaty on Principles Governing the Activities of States in the Exploration and Use of Outer Space, including the Moon and Other Celestial Bodies (OST) (18) contains many principles that apply to space resources utilization, including freedom of access to all areas, freedom of scientific investigation, nonappropriation, due regard, avoidance of harmful interference, and contamination. Yet, it did not establish an intergovernmental governing body, nor an associated set of rules and regulations to manage space mining activities.

We propose a Lunar Mining Code for inclusion into a future governance structure to manage water ice mining activities on the Moon. The mechanism includes a notification system for managing prospecting activities, a contract system for issuing exploration licenses to allotted areas on the Moon, best mining practices and principles to promote equal access, and area-based regulations to safeguard the lunar environment. We also propose a spatial planning tool such as the Lunar Mining Map Tool introduced by Hubbard (19) to implement the Lunar Mining Code. The tool defines resource systems where the Lunar Mining Code applies and divides that system into a grid of equal-sized blocks. The tool can also be used by the future governing authority to administer regulations and by the mining companies for supporting the proposed best mining practices.

## 1. The Deep Seabed and the ISA as Analogs for Managing Water Ice Mining on the Moon

Driven by challenges analogous to those on the Moon, the ISA established rules and regulations to manage seabed mining, thus becoming the only intergovernmental institution with established policies for managing mining activities in an Area Beyond National Jurisdiction (ABNJ). Regulations for prospecting and exploration have been adopted, though discussions are still ongoing related to exploitation. The deep seabed and lunar surface share many characteristics that permit using the ISA’s Mining Code as a framework for managing lunar water ice mining.

First, both are ABNJ. No nation, company, or person can exercise exclusive control over resources in ABNJ. If left ungoverned, ABNJs are susceptible to the tragedy of the commons (20), where appropriators could act in their own self-interest rather than the collective benefit of all. Because both locations are only accessible by nations with the necessary technological capabilities, the resources have the potential to be unevenly distributed, which could cause global economic imbalances and disputes.

Second, both the lunar surface and the seabed contain multiple mineral resources. While this paper is focusing on lunar water ice, each mineral resource will likely need its own set of unique regulations. The ISA’s Mining Code, for example, contains regulations for polymetallic nodules (21), massive sulfides (22), and cobalt-rich crusts (23), parallel in their scope and content. In addition to ice, the Moon’s regolith contains solar wind volatiles, ilmenite, anorthosite, volcanic glass beads, and other resources. Thus, the Lunar Mining Code will likely need to adopt a similar structure. However, as suggested by the Hague Space Resources Working Group (15), the governing authority should implement the evolutionary approach, developing regulations only when each resource is deemed economically viable (24). Moreover, limiting initial regulations to water ice allows the future governing authority to analyze the utility of existing regulations and adapt as needed.

Third, access to both the deep seabed and lunar surface is severely restricted due to their harsh environmental conditions. Such conditions require the development of bespoke, semiautonomous mining technologies tailored to their respective environments. The inaccessibility of both locations permits only operators possessing specialized technologies. Mining in these locations, however, has negative externalities. Seabed mining could permanently damage the marine environment and ecological diversity, while lunar mining activities could impact the scientific, cultural, and economic value of the Moon.

Fourth, both environments remain nearly completely unexplored. The limited knowledge of both locations and the potential impacts of human activities upon them indicates that humanity should exercise precaution and develop best mining practices to safeguard the lunar environment. In fact, because the OST (Art. III) (18) makes international law, including environmental law, applicable to the exploration and use of outer space, the precautionary principle (25) should be enforced by the future governing authority.

We use the ISA’s Mining Code and related literature (24–46) to formulate a preliminary Lunar Mining Code to manage lunar water ice mining activities.^*^ Antarctica was also considered since it is also an ABNJ. However, proposals introduced by Christopher Beeby to govern mining in the Antarctic (47, 48) ultimately led to the Madrid Protocol (49), which placed a prohibition on the extraction of mineral resources. While it is out of scope of this manuscript to conduct an in-depth analysis of other recommendations for governing mining on the Moon, we refer to previous research related to governance in ABNJ and of space resources in particular (14, 50–65) that should be considered for future iterations of a Lunar Mining Code.

## 2. The Lunar Mining Code

### 2.1. A Notification System to Manage Prospecting for Lunar Water Ice.

The first volume of the Lunar Mining Code addresses prospecting, the first phase in lunar water ice mining. Prospecting begins with a company delivering their equipment to an area of the lunar surface inferred to have elevated concentrations of water ice. Companies will then survey to determine the deposit’s composition, size, distribution, and accessibility at a relatively low resolution (e.g., at the 50 to 100 m scale). Like seabed mining, lunar prospecting campaigns will identify locations where ice is present in economic quantities. Thus, some form of coordination is needed. However, as mandated in Article 1(3) of the OST (18), the universal freedom to conduct scientific research on the Moon must be upheld. Policies are needed for safe and orderly operations, promoting equitable access, and to limit negative effects on the lunar environment. Thus, using the ISA’s Mining Code, we propose the following regulations in Table 1 to manage water ice prospecting activities.

**Table 1.** Volume 1 of the Lunar Mining Code: Regulations, procedures, and freedoms on prospecting for water ice at the Lunar South Pole

1. Operators do not receive exclusive rights over its prospecting area but can recover a “reasonable” quantity of resources necessary for characterization of the resource and resource environment.^⸸^
2. No spatial or temporal limits are imposed on prospecting.
3. Multiple actors can prospect within the same block, but must exercise due regard to the rights, duties, and freedoms of each other’s activities.^⸸^
4. Samples and data can be deemed proprietary to the operator.
5. Each operator must submit a notification to the managing authority of its intention to engage in prospecting, which includes information on a) the applicant and business, b) the launch, landing, deployment, and anticipated duration of the prospecting campaign, c) the equipment and methods expected to be used, d) the broad area where prospecting will be conducted (submitted in the form of blocks prescribed by the managing authority),^⸸^ and e) evidence of authorization by the appropriate state as per art. VI OST.
6. Each notification shall provide written undertakings that the operator will cooperate, comply, and accept as enforceable the adopted regulations and procedures set forth in the Lunar Mining Code.^⸸^
7. Operators shall take necessary measures to limit the effects of their mining activities on the lunar environment via the collection of environmental baseline data, the implementation of monitoring programs, and the submission of annual reports (See Section 2 for details).^⸸^
8. All mining equipment shall undergo decontamination to limit forward contamination.
9. All nonproprietary information submitted by the operator will be cataloged and made publicly available in a registry managed by the managing authority.
10. These regulations are to be interpreted and applied together with The Outer Space Treaty, The Rescue Agreement, The Liability Convention, and The Registration Convention.^⸸^

Regulations 1 and 2 in Table 1 align with Article I of the OST. Regulation 3 supports the due regard principle (OST, Article IX). While the ISA prohibits the recovery of minerals for commercial use, NASA vowed to purchase the rights of future moon samples recovered from private companies (66). Thus, Regulation 4 is proposed to support the development of the lunar economy. Regulations 5 and 6 provide a streamlined procedure to manage prospecting that promotes transparency. Information required in the notification process allows the managing authority to develop a comprehensive register of all mining assets on the lunar surface (Regulation 9). A registry and the disclosure of data help actors coordinate their activities, which promotes more orderly and safe operations.

The impact of mining activities on the lunar environment is uncertain. On Earth, regulatory oversight typically comes after the public recognizes the adverse impacts on the environment (67). The precautionary principle emphasizes taking early action in response to potential environmental threats, even in the context of scientific uncertainty (68). Yet rather than adopting regulations after irreversible environmental damages occur, Regulation 7 requires prospectors to collect environmental baseline data and implement monitoring programs. Annual reports are also recommended as a form of self-compliance and transparency. Regulation 10 aligns with existing international space law.

**Fig. 1.**
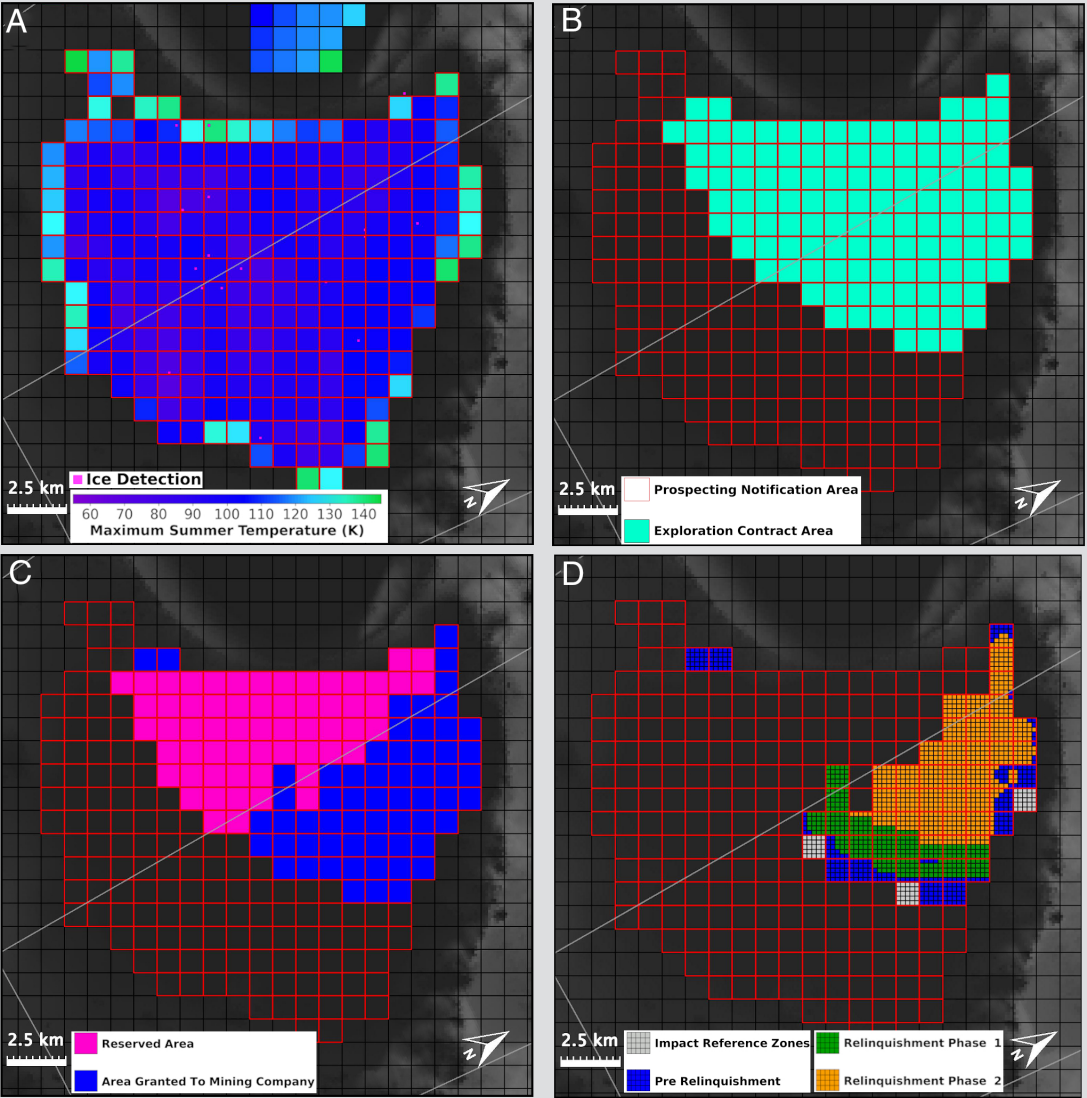
Depiction of a mining company’s steps to obtain exclusive rights to a water ice mine site on the Moon and how mining could be managed using the Lunar Mining Map Tool (19). The tool—overlaid on a composite base map consisting of a global morphology mosaic (69) and average percent illumination data (70)—defines the resource system and divides it into a grid of blocks. Plate (*A*) displays a mining company’s notification (blocks outlined in red) to the managing authority where they will conduct prospecting activities. The area (Nobile Crater) was determined by selecting blocks suitable for mining as defined by ref. 19, which are colored based on the average maximum temperature in the summer. Plate (*B*) displays a hypothetical exploration contract area submitted by a company. Plate (*C*) shows the division of the contract area into two hypothetical areas of equal commercial value determined using temperature and slope data. One is issued to the mining company (blue) and the other is deemed a Reserved Area (pink). In reality, multiple variables will be used for division and the contract area will likely be broken up into multiple clusters rather than two compact areas. Plate (*D*) shows the process of relinquishment and the establishment of Impact Reference Zones (gray blocks). Here, a mining company divided its exploration contract following the relinquishment protocol described in Section 2.3.2.

To initiate a prospecting campaign, a company first submits a notification containing the information under Regulation 5. The governing authority reviews the notification to ensure it is compliant with the regulations. Once approved, the blocks listed in the notification are cataloged in a register and available for other actors to see using a spatial planning tool (Fig. 1*A*). Doing so supports a level of transparency underlined in the Artemis Accords (13). The provision of prospecting areas is beneficial because it facilitates an operator’s capability to readily exercise due regard to others, which helps mitigate harmful interference. While disclosing IP provides a strategic advantage to other actors, the disclosure of preliminary prospecting data enables developing countries entry into an economy that would otherwise be monopolized by pioneering nations.

Moreover, consider the scenario where Mining Company A delivered prospecting equipment to a location on the Moon inferred to have water ice. Incomplete communication may lead other companies to prospect the same area, increasing the risk of interference and the eventual need for safety zones. Conversely, disclosure of prospecting data could help other mining companies identify similar geological or geophysical characteristics elsewhere on the lunar surface where water ice may be present. Rather than competing for the location, the disclosure of data provides an opportunity for safer operations within an ABNJ that is already extremely challenging.

### 2.2. A Contract System to Manage Water Ice Exploration Activities.

The second phase in lunar water ice mining is exploration, that is, the searching for resources under *exclusive rights*. Lunar exploration activities would include analysis of resources, use and testing of mining systems and facilities, and carrying out studies prior to exploitation to understand the effects of humans on the environment. In other words, this phase produces higher-resolution (e.g., 1 to 10 m scale) data about the deposit and determines whether the conditions are suitable for mining and whether companies can conduct these activities sustainably. To manage this step, we propose mining companies must submit a contract in the form of a plan of work for approval to a future intergovernmental institution. Upon approval, the applicant receives an exploration license for temporary, but exclusive jurisdiction (Section 2.2.1) over the mineral resources extracted from their contract area. The contract provides security of tenure, meaning that it cannot be suspended or amended unless the operator violates the terms of the contract (47, Annex IV, section 2).

Contracts are advantageous relative to statute-based governance approaches because they fill regulatory gaps in governing systems, create conditions or constraints on activities, and can require companies be held accountable for both anticipated and unanticipated environmental and social impacts of mining (71). Contracts also offer mutability, flexibility, and nimbleness, making them better equipped to create an enforceable regime (71) to govern mining on the Moon. Our proposed regulations on exploration for water ice at the Lunar South Pole are listed in Table 2 and depicted graphically in Fig. 1 *B*–*D*. The regulations would comprise the second volume of the Lunar Mining Code.

**Table 2.** Volume 2 of the Lunar Mining Code: Proposed regulations, procedures, and freedoms on exploration activities for water ice at the Lunar South Pole resource system

1. An approved plan of work grants a license for exclusive rights to conduct exploration activities to the blocks under contract.^⸸^ If another actor wishes to traverse through an area under contract, it must obtain explicit approval from the company and the managing authority before it does so, must demonstrate that there are no alternative pathways, and must exercise due regard to the assets within the contract area.
2. The operator must provide the same information listed in Table 1, Reg. 5 and exploration activities are also governed by Regulations 4,6-10 in Table 1.
3. The operator must also provide information on their exploration plans and the boundaries of the contract area, including a) the center coordinates of each block where exploration activities will be conducted, b) a map and prospecting data of the physical and geological characteristics of the contract area and the abundance, grade, and elemental contents of water ice, c) a general description and schedule of the exploration campaign, and d) a schedule of anticipated yearly expenditures for the first 10 y of exploration.^⸸^
4. An approved exploration contract is valid for no more than 25 y. Upon expiration, the operator can apply for a production contract or renounce their rights to the area covered in their contract (Section 2.2.1).^⸸^
5. The initial area granted to the operator shall be composed of no more than 120 mining blocks (~120 km2). Mining blocks must be arranged in clusters, where six contiguous blocks form a given cluster.^⸸^ The clusters shall be compact, proximate, and entirely located within the previously prospected area and cannot be arranged to deny access to any cluster of blocks not under contract.
6. Operators must adhere to the adopted policies, including a) the contribution of a Reserved Area (Section 2.3.1), b) the relinquishment protocol (Section 2.3.2), c) the establishment and use of Impact Reference Zones (Section 2.3.3), and d) adhering to regulations relating to Lunar Preservation Areas (Section 2.3.4).^⸸^
7. Each contract must contain information related to the financial and technical capabilities of the operator, including that is sufficient information to enable the managing authority to determine whether the applicant is financially and technically capable of carrying out its exploration plans.^⸸^

#### 2.2.1. Spatial and temporal regulations on exploration contracts.

Because exploration is a preparatory step when establishing a mine site, lunar water ice exploration contracts should only cover as large an area and last as long as needed for a company to identify a mine site. Thus, we propose the imposition of spatial and temporal constraints (Table 2, Reg. 4-5) on lunar exploration contracts. The constraints prevent monopolization of a resource system by pioneering investors and “lunar land grabbing” akin to what has been observed on Earth (72, 73).

While there is evidence that water ice is present at the lunar poles, the exact amount remains uncertain. Using lower and upper bounds of 0.5 wt % and 5.6 wt % (10) for water ice present in the upper meter of the lunar regolith and densities of ~1 t/m^3^ (74) and 1.65 t/m^3^ (75) for water ice and bulk lunar regolith, respectively, one mining block might contain ~9,000 to 98,000 t of ice. While the demand for lunar water ice is uncertain, Kornuta et al. (12) propose a near-term demand of 2,700 t/year of processed lunar water. From our idealized estimations, a company could fulfill this demand for ~3 to 40 y with one block. Yet because water ice will likely be unevenly distributed due to the variance in topographic and illumination conditions at the lunar poles, mining companies will likely need to explore more blocks. Although difficult to concretely determine, our high-level estimate suggests that a 120-block contract could sustain the demand in Kornuta et al. (12) for ~180 to 2,400 y. Thus, we recommend an applicant be limited to 120 blocks (Table 2, Reg. 5).

The ISA also requires blocks in exploration contract areas to be arranged in clusters (29, Annex II, section 19), though has not reported the rationale for this measure. We believe it is meant to constrain the sprawl of human activity and to reduce the effects of mining in ABNJ. Thus, we also propose an applicant to arrange blocks in their exploration contract in clusters (Table 2, Reg. 5). Care was taken in Reg. 5 to ensure that exploration contracts could not be used to prevent other actors from conducting operations in other nearby blocks.

### 2.3. Area-Based Management Regulations and Best Practices for Lunar Water Ice Mining.

Area-Based Management Tools regulate the distribution, timing, and intensity of activities in terrestrial ABNJ (76, 77). Each Area-Based Management Tool comprises a system of rights, measures, and procedures and can be designed to be 1) internationally, regionally, nationally, or independently managed, 2) static or dynamic in space and time, 3) completely or partially prohibitive, 4) applicable to entire regions or feature specific, and 5) sector-specific, multisectoral, or cross-sectoral. Four area-based management measures are proposed and described below for incorporation into the Lunar Mining Code.

In the oil and gas and seabed mining industries, exploration licenses are allocated to companies using a block system (78, 79). We recommend a future governing authority utilize a spatial planning tool such as the Lunar Mining Map Tool (19) to streamline management and interface with mining companies. The tool divides a given area on the lunar surface into a grid of blocks ~1 km × 1 km in size,^†^ establishing the resource system where the Lunar Mining Code applies; defining the boundaries of the resource system is an essential first step (80). In this example, the map tool defines the resource system as the Lunar South Pole from 80 to 90°S. The tool also standardizes the prospecting notification and exploration contract processes, streamlines how area-based measures are developed and implemented, facilitates rapid recognition of rights, enables enforcement and compliance of regulations and procedures, and can serve as a space registry’s graphic interface.

#### 2.3.1. Lunar reserved areas.

When a country submits an application to the ISA to conduct exploration activities for seabed mineral resources, they must divide their contract into two areas of equal commercial value. One area is granted to the applicant while the other is deemed a Reserved Area. Reserved Areas were developed to support the ISA’s “parallel system” that arose during negotiations. The Reserved Areas for use by developing states. We similarly propose that a mining company must contribute a Reserved Area from its contract area.

Because the Moon’s resources are available on a first-come-first-served basis, the first applicants will have a significant economic advantage. To promote equitable access, we propose that companies must identify two mine sites of equal commercial value, and both must have the appropriate environmental conditions to sustain a mine site. One area is granted to the company while the other is designated as a Reserved Area for a currently nonspacefaring nation. This policy is meant to lower the costs of exploration and reduce uncertainty around expected commercial revenue for developing countries (26, 33). Fig. 1*C* shows an example of a company dividing up their exploration contract area. First, the applicant applies for up to 120 blocks from their prospecting area (Fig. 1*B*). Prior to further exploration, the company provides a list of the block coordinates and sufficient prospecting data to delineate two suitable mine sites. Upon review and approval, the site is divided (Fig. 1*C*). The review and approval process with the governing authority is meant to ensure that the company is being equitable when dividing up its contract area.

Currently, the ISA allows entities from developed countries to sponsor a developing state by setting up shell companies (81). This permits a developed state to carry out exploration activities in Reserved Areas, which effectively grants them control of an area reserved for only developing states (82). To ensure that resources will be left for future spacefaring nations, we recommend Lunar Reserved Areas only be used in exploration contracts from applicant(s) from or sponsored by a developing state.

#### 2.3.2. Relinquishment.

Both the oil and gas industry (78, 79) and the ISA (35) have a process that allows “first movers” investing significant amounts of capital in mining to explore an adequate number of blocks to identify suitable exploitation sites, and then requires them to relinquish swaths of unused land that could then be used by future miners. The ISA’s relinquishment protocol was originally devised as a process for the first group of seabed mining applicants to divide up a resource-rich zone of the deep seabed (35). Because there were overlapping claims to this area, investors were required to progressively relinquish portions of the claim area until it was divided equally (35). The ISA has since adopted this to legally mandate companies to progressively relinquish portions of their contract area back to the Area (22, 23).

To prevent lunar land grabbing, we propose a relinquishment procedure for lunar mining exploration contracts. Similar to the ISA’s Mining Code, the procedure must be flexible, with the size, area, and schedule of relinquishment depending on the resource under contract (29). For water ice mining, we initially propose that if a company is granted 120 blocks, after 12.5 y of exploration, the mining company must relinquish 25% of its original contract area and after 25 y, at least 50% (Fig. 1*D*). The company is then granted priority rights to either apply for a commercial production license for the remaining contract area or renounce their rights. Entire blocks or individual subblocks can be relinquished, and relinquished blocks revert back to the resource system. The company must relinquish a block areas that are similar in shape and size (32). For the seabed, blocks were relinquished with classification schemes developed by each company (83). Similarly, each lunar water ice mining company will need to define its own methodology for relinquishment.

#### 2.3.3. Impact reference zones.

To conserve the marine environment, the ISA recommended contractors implement either Impact Reference Zones or use spatial management tools to monitor the impacts of their activities (32, 46). Future lunar mining operations can potentially contaminate the surface, suspend dust, alter the thermal environment, and release gases into the lunar exosphere (84, 85), which may impede communications with Earth, surface science operations, power generation, and thermal management. To reduce these effects, previous scholars called for international standards for extraterrestrial environmental assessment processes (67, 86, 87).

We propose companies follow the ISA model and designate Impact Reference Zones in their exploration contract areas where mining is prohibited (Fig. 1*E*). Impact Reference Zones would be monitored to collect environmental baseline data and to compare to blocks where mining is conducted. Monitoring programs will inform the creation of future policies on mining equipment and processes. Similar to measures within the BBNJ Agreement (88) and the ISA’s Mining Code (30), we propose that companies submit annual reports containing environmental impact statements, results of the efficacy of their monitoring programs, and what measures will be taken (if needed) to moderate adverse effects on the lunar environment. Thresholds for adverse environmental impacts would be defined by a Technical Commission appointed by the intergovernmental authority composed of experts from planetary science, sustainability, aerospace engineering, etc. The report would be synthesized and disseminated to the public by the governing authority.

A criterion must be formulated to determine the size and position of Impact Reference Zones. We recommend Lunar Impact Reference Zones cover ~5% of an operator’s contract area, include the targeted resource, and contain geologic and environmental settings similar to mining locations (e.g., illumination, temperature, etc.). The zones should be clustered in close proximity to facilitate monitoring, and their size, shape, and temporality should be highly flexible.

#### 2.3.4. Lunar preservation areas.

Celestial bodies need to be protected from human activities (89, 90). For example refs. 91 and 92 introduce the notion of Planetary Park Systems with extraterrestrial “wilderness policies” to limit access to these scientifically interesting regions. Such policies are necessary because 1) they would create a more complete and healthy concept of culture and civilization in space, 2) pristine land has intrinsic value, 3) extraterrestrial land should be protected for future generations, and 4) the land may contain things that are beneficial at some time in the future (91, 92). Ref. 93 calls for extending geoconservation principles to celestial bodies to identify geological and geomorphic features with scientific, historic, aesthetic, ecological, or cultural value. Moreover, ref. 94 promotes protecting the Moon from mining by establishing multipurpose nature reserves. Because the same water ice deposits being targeting for mining are of high scientific significance (95), we suggest the Lunar Mining Code include the potential to delineate Lunar Preservation Areas where water ice mining activities are prohibited.

Lunar Preservation Areas are akin to the ISA’s Areas of Particular Environmental Interest, which are a network of no-mining zones to protect a representative subset of ecosystems (52). The preservation areas were mandated to be established prior to the exploitation phase and designed to protect ~30 to 50% of the total mining area (96). Based on the applied principles for designing the ISA’s Areas of Particular Environmental Interest and previous work on planetary protection (89–94), we provide a list of potential design principles for Lunar Preservation Areas in Table 3, though these principles are preliminary and should be reviewed by the future intergovernmental authority.

**Table 3.** Preliminary design principles for Lunar Preservation Areas

1. Mining activities are prohibited in Lunar Preservation Areas.
2. To define a Lunar Preservation Area, a formal request would be submitted to the governing authority, which would approve the request based on a set of guidelines defined by the scientific and mining communities.
3. A predefined percentage of each Lunar Preservation Area must be agreed upon by the scientific community and protected before the exploitation phase of mining begins.^⸸^
4. The boundaries of Lunar Preservation Areas must be clearly defined using the block system and the center coordinates of each block should be delineated to the future governing authority.
5. Lunar Preservations Areas must be flexible, allowing for their numbers, locations, and sizes to be modified based on improved knowledge of the lunar environment and the impacts of mining activities.^⸸^
6. Lunar Preservation Areas should be designed as a configured network to conserve and preserve a full range and representative number of lunar features.^⸸^
7. Lunar Preservation Areas should be strategically positioned to ensure that the development of lunar resources is not inhibited, and the economic viability of the Moon is not depreciated.^⸸^
8. Access to Lunar Preservation Areas for scientific purposed shall be limited to predefined routes.

Lunar Preservation Areas could be defined and monitored using the same spatial planning tool described above (19). Scientists will need to determine what areas should be protected. To avoid resource depletion, Elvis and Milligan (97) argue that while economic growth remains exponential, humans should be limited to one-eighth of the exploitable materials in the Solar System, with the remainder seven-eighths being left as “space wilderness.” A simple alternative would be to require Lunar Preservation Areas be equal to the number of blocks licensed to operators. The benefit of this alternative is that as the number of exploration contracts increases, preservation areas also increase.

## 3. Concluding Remarks

We propose a Lunar Mining Code composed of area-based regulations to manage prospecting and exploration activities for water ice at the Moon’s poles. Elinor Ostrom—one of the most important thought leaders in governance in ABNJ—identified seven principles that make a common pool resource regime persist over time (98, p. 91). The Lunar Mining Code addresses three of these principles: 1) defined boundaries, 2) congruence between appropriation and provision rules and local conditions, and 3) monitoring. Our future work will detail the structure of the regime required to manage such work, which will address other principles noted in ref. 98, including enforcement, conflict-resolution mechanisms, collective-choice arrangements, and minimal recognition of rights to organize. We will also expand on the Lunar Mining Code to address how area-based management could be used to manage other phases in the water ice mining process (i.e., commercial production, clean-up, and remediation).

We believe the formulation of a governance system to manage mining on the Moon should progress now. Laws are needed to regulate mining activities, encourage the sustainable development of lunar resources, safeguard the lunar environment, and ensure responsible and equitable use of the Moon’s resources.

If the Moon is left ungoverned, the first lunar mining companies would be incentivized to extract unnecessary amounts of resources. Similar to deep sea mining (99), if states opt for multilateral agreements over a single intergovernmental regime or choose to follow only domestic legislation, minitreaties between states would likely materialize. This could lead to the emergence of multiple governing bodies and the potential overlapping of claims over resource-rich areas (100). Such events could increase disputes and create confusion over rights. We hope the Lunar Mining Code will serve as a foundation for the international community to ensure such issues will not arise as the lunar space economy continues to develop.

## Data Availability

Some study data are available and have been deposited in the Harvard Database (https://dataverse.harvard.edu/dataset.xhtml?persistentId=doi:10.7910/DVN/Q1Y3OS). These data include files in the formats of .csv, .txt, .jlf (JMars file), .prj, and .tif. Previously published data were used for this work (19).

## Footnotes

^*^Numerous regulations, best practices, and principles in the Lunar Mining Code were synthesized, paraphrased, rewritten, and reformulated from the ISA’s Mining Code. These are marked in this manuscript with⸸ symbol.

^†^Because the grid was developed using a stereographic projection, blocks vary slightly in size since distortion of area and distance increases away from the center of projection. However, there is only ~1.5% difference between the smallest (0.987 km2) and largest (1.002 km2) grid block. The grid’s shape was prioritized since the use of straight lines is a fundamental principle in the design of terrestrial area-based management tools (101).
